# Liver Fibrosis and Cirrhosis in Patients with Non-alcoholic Fatty Liver with and without History of Cholecystectomy: A Pilot Study

**DOI:** 10.34172/mejdd.2024.366

**Published:** 2024-01-31

**Authors:** Abazar Parsi, Eskandar Hajiani, Somayeh Sadani, Seid Jalal Hashemi, Seid Saeed Seyedian, Mehdi Alimadadi, Reza Ghanbari

**Affiliations:** ^1^Alimentary Tract Research Center, Clinical Sciences Research Institute, Imam Khomeini Hospital, Ahvaz Jundishapur University of Medical Sciences, Ahvaz, Iran; ^2^Clinical Research Development Unit (CRDU), Sayad Shirazi Hospital, Golestan University of Medical Sciences, Gorgan, Iran; ^3^Gene Therapy Research Center, Digestive Diseases Research Institute, Tehran University of Medical Sciences, Tehran, Iran

**Keywords:** Non-alcoholic fatty liver disease, Liver fibrosis, Elastography, Cholecystectomy

## Abstract

**Background:**

Non-alcoholic fatty liver disease (NAFLD) is one of the most common chronic liver diseases in the world. Previous studies revealed that cholecystectomy may be considered a risk factor for the development of NAFLD. The aim of this study was to compare the amount of liver fibrosis, determined by elastography, between patients with NAFLD with and without a history of cholecystectomy.

**Methods:**

In this descriptive-analytical cross-sectional study, 50 patients with NAFLD were divided into two groups: one with a history of cholecystectomy and the other without. No significant differences were found between these two groups in terms of age or sex distribution. Liver fibrosis was measured for all patients using an elastography imaging system. Subsequently, the data related to liver fibrosis, along with the demographic information of the patients, were statistically analyzed using SPSS software version 22.

**Results:**

The mean elastography score in all patients was 10.66±12.18 kPa (the elasticity scale ranging from 3.80 to 66.40 kPa). The group with a history of cholecystectomy had a significantly higher mean elastography score (13.39±16.20 kPa) compared with the group without cholecystectomy (7.93±4.99 kPa) (*P*=0.02). Additionally, there was a significant positive correlation between body mass index (BMI) and the mean elastography score in the group of patients with a history of cholecystectomy.

**Conclusion:**

The mean elastography score of patients with NAFLD with a history of cholecystectomy was approximately twice as high as that of non-cholecystectomy patients.

## Introduction

 Non-alcoholic fatty liver disease (NAFLD) is one of the most common chronic liver diseases worldwide. This disease is associated with hepatic steatosis, elevated levels of triglycerides, liver enzymes, and several inflammatory biomarkers.^1,2^ NAFLD encompasses a range of liver disorders, from simple steatosis to liver fibrosis and cirrhosis. If left untreated, these conditions can eventually lead to liver cancer and death. NAFLD is commonly linked to metabolic syndrome disorders such as obesity, insulin resistance, hypertension, dyslipidemia, and lipid metabolism disorders. These factors increase the risk of cardiovascular disease-related mortality.^3,4^

 The prevalence of NAFLD is increasing due to urbanization and lifestyle changes, affecting approximately 20-30% of the general population.^5^ Currently, around 25% of Asian individuals have some degree of fatty liver.^6^ In the Iranian adult population, the reported prevalence ranges from 21.5% to 31.5%.^7^ The increasing rates of obesity and overweight contribute to the rising prevalence of NAFLD.^8,9^ NAFLD and gallbladder diseases, following cholesterol gallstones, are conditions that can impact overall societal health. Both diseases are prevalent among adults, with an incidence rate ranging from 10% to 50% in patients with cholesterol gallstones in the United States and Europe.^10-12^

 The results of previous studies have shown that cholecystectomy can be considered a risk factor for the development of NAFLD.^13^ The metabolic effects resulting from the absence of gallbladder after cholecystectomy can ultimately contribute to the development of NAFLD.^14^ Furthermore, previous studies have demonstrated an association between cholecystectomy and liver cirrhosis, as well as an increase in serum levels of liver enzymes.^15^ However, other studies with a large sample size have shown no relationship between fatty liver disease and cholecystectomy.^16^ While an extensive study in Asia concluded that cholecystectomy could significantly increase the incidence of NAFLD,^17^ these differing results emphasize the need for further research in this field. In chronic liver diseases, accurately estimating the severity of liver fibrosis and evaluating anti-fibrosis treatments requires precise variables commonly known as fibrotic markers.^18,19^

 Liver biopsy is still considered the gold standard procedure to evaluate inflammatory activities and liver fibrosis despite being an invasive method with potentially serious complications. However, performing serial liver biopsies to assess treatment response within a short period is impractical and cost-effective. As a result, numerous researchers have recently proposed non-invasive experiments, including hybrid or single-line tests, as a viable alternative to biopsy.^20,21^ FibroScan or transient elastography (TE) is a novel method used to assess liver fibrosis. It utilizes ultrasound technology to measure liver stiffness and estimate the degree of fibrosis.^22,23^ Considering the challenges associated with liver biopsy, including its invasive nature and potential complications, FibroScan offers a scientific, accurate, and non-invasive alternative for the development of diagnosis and research in the Future.^24^ Studies have also introduced transient elastography methods to identify cirrhosis and fibrosis grade F2 and higher with high sensitivity and specificity.^25^

 Given the limited availability of elastography in certain centers and the lack of economic benefits associated with it, relatively few studies have been conducted using this diagnostic method. Additionally, no research has been done to investigate the history of cholecystectomy as a potential marker for further fibrosis development in patients with fatty liver. It is evident that liver complications, such as weakening following gallstone formation in the liver, are possible after cholecystectomy.^10^ The primary objective of this study was to compare the extent of liver fibrosis between patients with NAFLD with and without a history of cholecystectomy using an elastography procedure.

## Materials and Methods

###  Study Population

 Given the absence of comparable studies exploring elastography in patients with fatty liver with or without a history of cholecystectomy, we conducted a pilot study to address this research gap. In the present descriptive-analytic cross-sectional study, after obtaining permission from the Ethics Committee of Ahvaz Jundishapur University of Medical Sciences (IR.AJUMS.REC.1397.084) and seeking statistical consultation, we enrolled 50 patients with NAFLD who provided written informed consent. According to their medical histories, the patients were divided into two groups: those with a history of cholecystectomy (25 patients) and those without a history of cholecystectomy (25 patients).

 The inclusion criteria for this study consisted of patients who were diagnosed with NAFLD and were over 18 years old. Additionally, in the group with a history of cholecystectomy, at least one year had passed since their cholecystectomy operation (Table 1). On the other hand, the following were considered exclusion criteria: daily alcohol intake exceeding 20 g, advanced liver disease, alanine transaminase (ALT) levels above 120 units per liter (U/L), liver dysfunction or any liver mass present, viral and autoimmune hepatitis, history of gastrointestinal surgery (excluding cholecystectomy), history of prolonged intravenous feeding within the last 6 months, use of hepatotoxic drugs during that period as well as Wilson’s disease, hemochromatosis, and heart failure.

**Table 1:** Demographics markers and elastography parameters in two groups

**Variables**	**Cholecystectomy**		**Non-cholecystectomy**		***P***** value**
	**Mean**	**SD**	**Mean**	**SD**	
Age	45.0800	15.15454	38.7600	9.58853	0.08
Height	169.2400	10.32909	173.3600	7.65332	0.11
Weight	83.1200	18.97217	84.1800	12.42551	0.81
BMI	29.0777	6.58609	28.0151	3.85956	0.49
ALT	47.4286	26.33168	68.4583	31.82285	0.02 *
Elastography	13.3920	16.20974	7.9320	4.99581	0.02 *

 In this study, we assessed the average duration of cholecystectomy among the enrolled patients until the time of elastography. The mean duration was 3.93 ± 4.79 years, with a range spanning from 3 months to 20 years. Furthermore, our analysis did not identify any statistically significant correlation between the variable duration of cholecystectomy and the average elastography results (*P* = 0.51).

 The major criteria defining metabolic syndrome encompass central obesity, elevated blood triglyceride (TG) levels, reduced high-density lipoprotein cholesterol (HDL-C) levels, increased blood sugar levels, and hypertension. These factors collectively contribute to the diagnosis of metabolic syndrome and provide valuable insights into an individual’s metabolic health.^26^

###  Liver Fibrosis Measurement by Elastography

 A total of 25 patients in each group (with and without a history of cholecystectomy) underwent elastography to determine the extent of liver fibrosis. After assessing patients for inclusion criteria and grouping them based on their cholecystectomy history, data related to fatty liver and underlying diseases, as well as demographic information, including age, sex, height, and weight (for body mass index [BMI]), were recorded. Subsequently, all enrolled patients were referred to the elastography center for liver fibrosis measurements using elastography.

 The cut-off values for detecting significant stiffness in elastography were determined as F4 > 10.3 and F3 > 8.7, while the cut-off value for cirrhosis was set at 11.5 kPa.^27^ In the current study, liver fibrosis analysis was performed using the EchoSens FibroScan device (Model 502, France). Finally, elastography data, along with demographic information and patient database, were collected for further analysis.

 Endoscopic ultrasound (EUS) and magnetic resonance cholangiopancreatography (MRCP) are commonly employed for the examination of bile ducts and pancreas.^28^ While EUS and MRCP play crucial roles in certain clinical scenarios involving biliary or pancreatic pathologies, we did not include them as part of our study due to their limited applicability for evaluating liver fibrosis accurately.

###  Statistical Analysis

 After collecting the statistical findings, a comparative analysis of the results was conducted using SPSS software version 22. The chi-square test was used to compare qualitative variables between the two groups, while an independent t-test was employed to compare quantitative variables, assuming a normal distribution of data.

## Results

 Out of the 50 patients participating in this study, 56% were men (28 out of 50). The mean age of patients with NAFLD with a history of cholecystectomy was 45.08 ± 15.15 years, while in the group without cholecystectomy, the mean was 38.76 ± 9.58 years. There was no statistically significant difference between the two groups in terms of age (*P* = 0.08). Statistical analysis also revealed that there were no significant differences between the two groups in terms of height (*P* = 0.11), weight (*P* = 0.81), and BMI (*P* = 0.49).

 When comparing the duration of the NAFLD, it was found that 13 patients (26.5%) had a disease history of less than 6 months, 20 patients (40.5%) were affected between 6 months and 1 year, 13 patients (26.5%) had a history of more than one year, and three patients (6.1%) were recently diagnosed with this disease. It was observed that the duration of the disease was significantly longer in the group with a history of cholecystectomy (*P* = 0.01). In addition, the mean ALT level in the cholecystectomy group was significantly higher (*P* = 0.02).

 The mean elastography score in all studied patients was 10.66 ± 12.18 kPa, with a range of 3.80 to 66.40 kPa. Comparative analysis of the two groups revealed that the mean elastography score in patients with a history of cholecystectomy was significantly higher compared with those without cholecystectomy (*P* = 0.02). Regarding the grades of non-alcoholic fatty liver based on ultrasound results, it was found that five patients were classified as grade 1, while 41 patients were grade 2, and only three patients were grade 3. There was no statistically significant difference in grade between the two compared groups (*P* = 0.76). Furthermore, in terms of liver fibrosis and cirrhosis comparison, among the cholecystectomy group, 15 patients had less than F3 fibrosis, four patients had features of F3, one patient was diagnosed with grade F4, and five patients were considered as having cirrhosis. Also, in the group without a history of cystectomy, 20 patients were less than F3, two patients had a fibrosis score of F3, and three patients had cirrhosis. There was no significant difference between the two groups regarding liver fibrosis and cirrhosis (*P* = 0.41). Although the overall elastography scores differed among the patients, the number of patients exhibiting different degrees of fibrosis and cirrhosis in the two groups was not statistically significantly different.

 Our findings indicated that in the group of patients with a history of cholecystectomy, there were no significant correlations between age, weight, disease duration, ALT level, and cholecystectomy time with elastography scores. However, a significant negative correlation was observed between patients’ height and elastography scores. This means that patients with shorter height had higher elastography scores. Furthermore, a significant positive correlation was found between patients’ BMI and the elastography scores; as BMI increased, so did the elastography score. In contrast, data analysis for patients without a history of cholecystectomy revealed no significant correlations between any of the mentioned variables and elastography scores

## Discussion

 Cholecystectomy may contribute to the development of NAFLD by altering the circulation of bile acids (BA), activating BA receptors, and disrupting signaling pathways related to hepatic lipid and glucose metabolism. Additionally, it has been demonstrated that the reduction in fibroblast growth factor 19 (FGF-19) levels in patients with cholecystectomy can increase liver triglyceride content, potentially influencing the development of NAFLD.^17^ This study is one of the few that compares liver fibrosis using elastography in patients with NAFLD, both with and without a history of cholecystectomy. Based on similar studies conducted in this field, it has been established that transient elastography can be considered a non-invasive and powerful method for identifying liver fibrosis. However, only limited research has been conducted on this topic thus far.

 In a study conducted by Hormati et al on 60 patients, which aimed to assess the diagnostic value of non-invasive methods for identifying liver fibrosis, comparisons were made between elastography, BARD (BMI, AST/ALT ratio, diabetes) scores, and BAAT (BMI, ALT, age and triglycerides) scores. The results demonstrated that elastography could serve as a robust tool for diagnosing liver stiffness and cirrhosis.^29^ Another comprehensive study by Hajiani and colleagues, comparing transient elastography findings with liver biopsy in patients with chronic liver disease, found a significant association between the stage of fibrosis and the degree of liver stiffness. Additionally, they reported a significant correlation between mean liver stiffness measurement and the stage of fibrosis.^30^

 In our study, we evaluated the demographic variables of patients and their relationship with diagnostic outcomes. The BMI of patients with a history of cholecystectomy was identified as a risk factor associated with diagnostic results. This variable was also analyzed in a study by Wang et al, where they examined the prevalence of fatty liver and advanced fibrosis. They reported that transient elastography demonstrated better fibrosis detection in patients with fatty liver and lower BMI.^31^ Their findings suggested an easier diagnosis in patients with low BMI, while our present study concluded that lower BMI was associated with less liver stiffness. Both studies indicate that elastography can be used for diagnosing disease in patients with more fibrosis. Furthermore, Sandrin and co-workers, in their study involving 106 patients with chronic hepatitis C, investigated liver fibrosis using FibroScan or transient elastography. They observed that this non-invasive method was useful for detecting the extent of liver fibrosis and could accurately identify fibrosis beyond F2 as well as cirrhosis. Moreover, their results were reproducible.^32^

 The main objective of the present study was to assess the impact of cholecystectomy on fibrosis and its diagnosis using elastography. Ultimately, no significant difference in liver fibrosis was observed between patients with and without a history of cholecystectomy. Similar findings were reported in other studies where cholecystectomy was not identified as a significant risk factor for liver disease. In a large retrospective study conducted by Wang et al, which reviewed medical records and abdominal ultrasounds of over 30 000 patients, it was concluded that cholecystectomy did not significantly increase the risk of developing fatty liver disease.^16^ However, contradictory results have also been reported, highlighting the need for further research with expanded variables. In another cross-sectional study involving more than 17 000 patients conducted by Kwak and colleagues, multiple regression analysis demonstrated a significant correlation between cholecystectomy and NAFLD but no significant association with gallstones. Consequently, they suggested that cholecystectomy might be a risk factor for NAFLD.^17^ Additionally, Ioannou found in a related study that the incidence of hospitalization or death due to cirrhosis is approximately two times higher in patients with a history of cholecystectomy. This led them to conclude that cholecystectomy could serve as a predictor for the development of cirrhosis.^15^

 Our study successfully evaluated patients across different ranges of liver stiffness and diagnosed liver cirrhosis using the non-invasive elastography method in both patients with NAFLD with and without cholecystectomy. However, further research is still needed to fully comprehend the relationship between cholecystectomy, fatty liver, and the degree of liver fibrosis. To obtain more comprehensive results, it is recommended to eliminate confounding variables and compare elastography with other diagnostic methods for analyzing liver fibrosis and cirrhosis. It is worth noting that our present study was conducted as a pilot study on a small number of patients with limited follow-up time due to the absence of similar studies in this field. Based on these preliminary findings, future larger-scale studies can be designed with longer follow-up periods. This will allow for more robust conclusions regarding the relationship between cholecystectomy, fatty liver, and liver fibrosis by including a greater number of participants.

## Conclusion

 In conclusion, our study revealed that patients with NAFLD with a history of cholecystectomy had significantly higher mean elastography scores, longer duration of NAFLD, and elevated ALT levels compared with those without a history of cholecystectomy. However, we did not observe any significant differences in liver stiffness grade, fibrosis, or cirrhosis between these two groups. Furthermore, in patients with a history of cholecystectomy, there was a positive correlation between BMI and elastography score and a negative correlation between height and elastography score.
